# Identification and elimination of genomic regions irrelevant for magnetosome biosynthesis by large-scale deletion in *Magnetospirillum gryphiswaldense*

**DOI:** 10.1186/s12866-021-02124-2

**Published:** 2021-02-25

**Authors:** Theresa Zwiener, Frank Mickoleit, Marina Dziuba, Christian Rückert, Tobias Busche, Jörn Kalinowski, Damien Faivre, René Uebe, Dirk Schüler

**Affiliations:** 1 Department of Microbiology, University of Bayreuth, Bayreuth, Germany; 2 Institute of Bioengineering, Research Center of Biotechnology of the Russian Academy of Sciences, Moscow, Russia; 3 Center for Biotechnology, University of Bielefeld, Bielefeld, Germany; 4 Department of Biomaterials, Max Planck Institute of Colloids and Interfaces, Potsdam, Germany; 5 Aix-Marseille Université, CEA, CNRS, BIAM 13108, Saint Paul lez Durance, France

**Keywords:** *Magnetospirillum gryphiswaldense*, Magnetosomes, Genome reduction

## Abstract

**Background:**

Magnetosome formation in the alphaproteobacterium *Magnetospirillum gryphiswaldense* is controlled by more than 30 known *mam* and *mms* genes clustered within a large genomic region, the ‘magnetosome island’ (MAI), which also harbors numerous mobile genetic elements, repeats, and genetic junk. Because of the inherent genetic instability of the MAI caused by neighboring gene content, the elimination of these regions and their substitution by a compact, minimal magnetosome expression cassette would be important for future analysis and engineering. In addition, the role of the MAI boundaries and adjacent regions are still unclear, and recent studies indicated that further auxiliary determinants for magnetosome biosynthesis are encoded outside the MAI. However, techniques for large-scale genome editing of magnetic bacteria are still limited, and the full complement of genes controlling magnetosome formation has remained uncertain.

**Results:**

Here we demonstrate that an allelic replacement method based on homologous recombination can be applied for large-scale genome editing in *M. gryphiswaldense*. By analysis of 24 deletion mutants covering about 167 kb of non-redundant genome content, we identified genes and regions inside and outside the MAI irrelevant for magnetosome biosynthesis. A contiguous stretch of ~ 100 kb, including the scattered *mam* and *mms6* operons, could be functionally substituted by a compact and contiguous ~ 38 kb cassette comprising all essential biosynthetic gene clusters, but devoid of interspersing irrelevant or problematic gene content.

**Conclusions:**

Our results further delineate the genetic complement for magnetosome biosynthesis and will be useful for future large-scale genome editing and genetic engineering of magnetosome biosynthesis*.*

**Supplementary Information:**

The online version contains supplementary material available at 10.1186/s12866-021-02124-2.

## Background

Besides their function as magnetic sensors and importance as models for prokaryotic organelle biosynthesis, magnetosomes formed by magnetotactic bacteria represent magnetic nanoparticles that are highly attractive for several biotechnological and biomedical applications [1–3]. Because of its tractability and relatively straightforward cultivation, the alphaproteobacterium *Magnetospirillum gryphiswaldense* has emerged as a model for studying the biosynthesis of magnetosomes, as well as their bioproduction and engineering for various applications [4–11]. Magnetosomes isolated from *M. gryphiswaldense* are composed of monocrystalline cuboctahedral crystals of magnetite (Fe_3_O_4_) about 35 nm in size, which are enveloped by a protein-lipid membrane [12]. In the biotechnological and biomedical field, they have been studied, for instance, as nanocarriers for magnetic drug targeting [13–15], multimodal reporters for magnetic imaging [16, 17], and for magnetic hyperthermia applications [18, 19]. In addition, the functionality of magnetosomes can be greatly extended by engineering the magnetite crystals and genetic coupling of magnetosome membrane proteins to foreign functional moieties such as fluorophores, enzymes, antibodies, and organic shells [6, 7, 20–24].

The exquisite properties of magnetosomes, such as high chemical purity and crystallinity, strong magnetization, uniform shapes and sizes [25] are due to the strict control over their biomineralization. This is orchestrated by more than 30 biosynthetic genes, which were mostly found to be clustered in a single chromosomal region, the genomic **ma**gnetosome **i**sland (MAI) [26–29]. The MAI harbors the polycistronic operons *feoAB1op*, *mms6op*, *mamGFDCop*, *mamABop*, and *mamXYop*, which control all specific steps of magnetosome biosynthesis such as the formation of intracellular membrane vesicles, the uptake of iron, magnetite biomineralization, and the assembly of the magnetite crystals into well-ordered chains [3]. The five key operons are separated by stretches containing genes of yet unknown, but irrelevant function for magnetosome biosynthesis [29]. These intervening MAI regions harbor numerous mobile genetic elements, repeats and genetic “junk” (e.g., several incomplete and pseudogenes as well as non-coding genetic content), which are thought to be responsible for genetic instability, i.e., frequent rearrangements, deletions and the spontaneous loss of the magnetic phenotype during subcultivation of *M. gryphiswaldense* [26, 27, 30]. For future genetic analysis and manipulation of magnetosome biosynthesis, it would therefore be highly desirable to eliminate and replace these regions by a compact cassette comprising only the essential biosynthetic gene clusters, but devoid of genetic junk. Mutagenesis by several large, overlapping deletions of up to 61 kb has already demonstrated that a total of 115 kb of the MAI can be eliminated without any detectable effects on growth and magnetosome formation [28, 29]. However, the role of distal and MAI-adjacent regions remains unclear.

Recently, reverse and forward genetic approaches suggested that, besides the well-established *mam/mms/feo* operons within the MAI, there might be further, auxiliary determinants for magnetosome biosynthesis encoded somewhere else in the genome. For example, a genome-wide transposon mutagenesis screen revealed numerous hits outside the MAI [31], however, the putative involvement of several of the afflicted genes still has to be verified by their clean deletions. In addition, a comprehensive proteomic analysis of the magnetosome membrane revealed several novel genuine constituents [32]. However, their putative roles in magnetosome biosynthesis also still await confirmation by deletion mutagenesis of respective genes.

Large-scale genome analysis and editing in magnetic bacteria would greatly benefit from efficient and reliable techniques for large genetic deletions. For the excision of fragments up to ~ 53 kb a Cre-*lox* based method has been used [28, 29] in *M. gryphiswaldense*. However, this technology has several practical disadvantages, as it requires the cumbersome construction and insertion of two different vectors with *lox* sequences integrating by homologous recombination upstream and downstream of the target region and carrying two different antibiotic resistances. An additional helper plasmid encoding the *Cre* recombinase needs to be conjugated into the host to induce excision of the targeted chromosomal segment, and finally has to be cured from the deletant. In addition, *loxP* nucleotides remain in the genomic target region, causing so-called scars [28]. Alternatively, an allelic replacement method based on homologous recombination has been routinely used for scarless deletions in *M. gryphiswaldense* [33], requiring only one vector, and taking advantage of counterselection of the vector excision by double-crossover using the suicide gene *galK* that encodes a galactokinase with lethal activity [34]. However, this method so far has been employed only for the deletion of smaller fragments (< 20 kb), but not tested for the excision of larger regions.

In this study, we first tested gene deletion methods available for *M. gryphiswaldense* with respect to their practicability and performance in large-scale mutagenesis and engineering. Next, by systematic deletion analysis of the extended MAI as well as adjacent chromosomal regions we interrogated their relevance for magnetosome biosynthesis and growth under lab conditions. Identified irrelevant gene content was substituted by a compact version of all key biosynthetic gene clusters, thereby eliminating much ‘junk’ and putative detrimental gene content. In addition, further candidate genes outside the MAI that had been putatively implicated in magnetosome biosynthesis by previous reverse and forward genetic approaches were probed by targeted deletions [31, 32].

## Results

### Evaluation of the large-scale deletion method

**Fig. 1:**
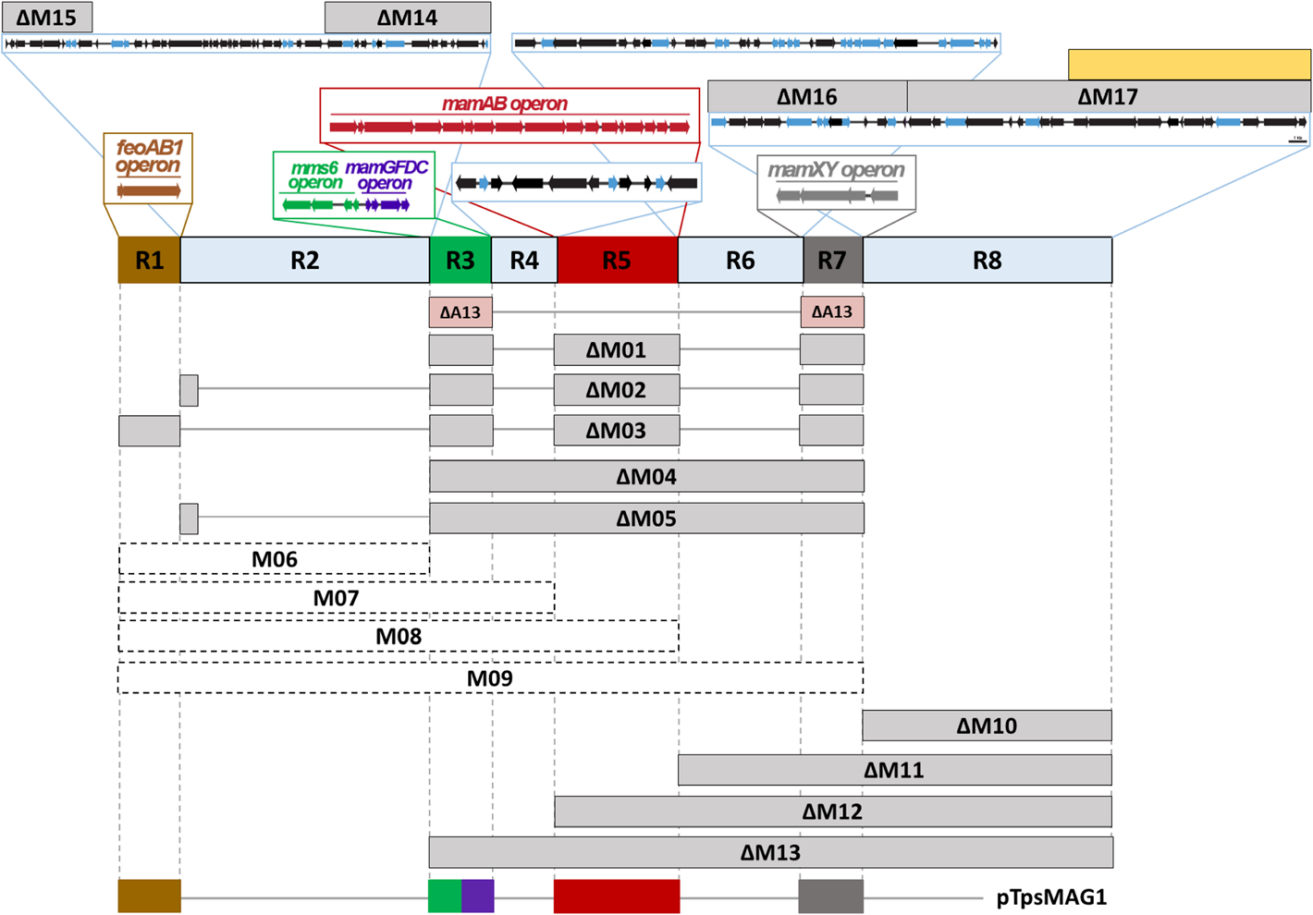
Overview over generated MAI and MAI-adjacent mutants in *M. gryphiswaldense*. Regions R1, R3, R5 and R7 indicate the five key operons (brown, green, violet, red, grey) for magnetosome biosynthesis while R2, R4, R6 and R8 represent intervening and MAI-adjacent regions. Grey bars show the extensions of successful deletions, while connecting lines indicate non-deleted parts. Magenta color highlights the strain ∆A13 from Lohße et al. (2011) which served as parental strain for ∆M01 and ∆M03. Dashed bars show attempted deletions which failed. Genes with irrelevant functions for magnetosome biosynthesis are shown as black arrows, and transposable elements are shown in blue. The yellow bar indicates the extent of deletion (~ 12.8 kb) that had not been covered by previous approaches [28, 29]. The genotype of the five contiguous magnetosome biosynthesis operons compact cassette pTpsMAG1 is shown in the lower line containing regions R1, R3, R5 and R7 (brown, green, violet, red, grey)

We first assessed two different techniques with respect to their usability and efficiency to introduce large genomic deletions: A Cre-*lox* based method, which had been used for excision of larger fragments before [28, 29, 35], and an allelic replacement method based on two consecutive double-crossovers counterselected by lethal GalK [34]. These were tested on two different regions (∆M01/~ 16 kb, and ∆M04/~ 66 kb) of the MAI (Fig. 1).

**Fig. 2:**
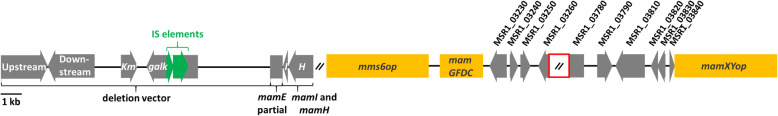
Results of genome re-sequencing of a typical false positive clone isolated during attempts to delete a ~ 68 kb region (M08). The red box indicates an unintended deletion of ~ 47 kb located between MSR1_03260 and MSR1_03780 (nt position 360,736). Brackets indicate parts of the remaining suicide vector with the Km^r^ marker and the *galK* gene inactivated by a spontaneous insertion of a tandem IS element (green arrows), as well as an unintended insertion of several *mam* genes (*mamH*, *mamI* and parts of *mamE*) next to the remnants of the suicide vector

By using the Cre-*lox* based method, plenty of clones containing the desired ∆M01 and ∆M04 deletions could be isolated (typically around 20–30 clones with excised target regions per 96 screened clones). Using allelic replacement, between 15–30 clones with the desired double-crossover were typically obtained from 96 screened clones after the final counterselection step. As expected, we found that the use of longer homologous regions of about 1.5–2.5 kb is favorable to yield high numbers of positive clones for larger (>ca. 20 kb) deletions, whereas fragments larger than 2.5 kb were difficult to clone by overlap PCR. Excluding time for cloning, Cre-*lox* based deletions in our hands typically required about 6 weeks because of the need of three consecutive cycles of laborious conjugation, plate growth, clonal selection and screening, which are particularly cumbersome and time-consuming in the rather slow-growing *M. gryphiswaldense*. In contrast, after some streamlining of the workflow, by GalK selection and double-crossovers a clean unmarked deletion mutant was typically obtained and PCR-verified in only about 3 weeks. During this study, this method later also proved to be highly efficient for deletions of up to about 100 kb. While in most cases proper excisions by double-crossovers could be confirmed by sequencing of PCR products spanning over the excision site, occasionally we identified clones which yielded amplicons of expected size, but did not have lost their insensitivity against kanamycin, indicating the Km^r^ marker harbored on the suicide vector to be still residing in the genome. This issue is exemplified by a clone in which we had attempted a ~ 68 kb deletion spanning from *feoAB1op* to *mamABop* (region M08, see below and Fig. 2). Despite their kanamycin insensitivity, all cells had apparently lost the ability to form magnetosomes as expected. However, genome resequencing revealed a large part (~ 44 kb) of the deletion target to be still residing in the chromosome, and a large part (~ 11.4 of ~ 11.7 kb) of the suicide vector was inserted next to it. Conspicuously, the orientation of the homologous downstream region had become inversed, and a ~ 2 kb fragment of *mamABop* (comprising *mamH*, *mamI* and a part of *mamE*) was dislodged from its native position, while the rest of this operon (including several essential magnetosome genes) was absent (Fig. 2), thereby explaining the loss of the magnetic phenotype. Likely, deletion of ~ 47 kb exceeding targeted M08 region had occurred by homologous recombination between two nearly identical ~ 750 bp stretches of two integrase genes residing in R4 and R6, respectively.

Notably, in this clone we also found the suicide gene *galK* (encoding the lethal galactokinase) to be inactivated by insertion of IS elements, thereby prohibiting proper counterselection in the presence of galactose, but favoring the occurrence of spontaneous homologous and non-homologous rearrangements instead. Similarly, during the further course of our mutagenesis approach, false positive clones instead of the intended ‘clean’ deletions were frequently obtained, in particular for difficult or essential targets. Resequencing of all such suspicious clones revealed that this was always accompanied by *galK* inactivation due to IS insertions (Fig. 2). Nonetheless, considering the benefits of the GalK-based method, it was chosen for all subsequent deletions in this work.

### Deletion and replacement of the MAI and adjacent regions

We next generated a library of strains in which we aimed to delete all key magnetosome biosynthesis genes plus as much as possible of the interspacing and flanking gene content from the ~ 100 kb MAI [3]. This region is known to be particularly rich in genetic junk and comprises 39 putative mobile genetic elements [26–29] (Fig. 1, blue arrows). We genetically dissected the MAI and its neighboring region for testing their relevance regarding survival, cell growth and magnetosome biosynthesis. By excluding genes assumed to be relevant or essential for cell growth (e.g. tRNAs and rRNAs), we predicted a region of ~ 134 kb comprising all known key magnetosome clusters and genes potentially irrelevant to the magnetosome formation (Fig. 1), including region R2 that seemed to be successfully deleted in Ullrich et al. (2010), while it appeared to be non-deletable in Lohße et al. (2011). The whole ~ 134 kb region was divided into eight separate regions (R1–8) representing putative deletion targets, which comprised known magnetosome biosynthesis operons (R1, R3, R5, R7), intervening regions (R2, R4, R6) and a flanking region adjacent to the MAI (R8). Since regions R2 and R8 are spanning large chromosomal areas containing many hypothetical genes with unknown function, they were further divided into smaller parts for deletion. In summary, all regions were covered by 17 partially overlapping deletion targets spanning from ~ 2.5 kb (*feoAB1op*) up to ~ 100 kb (∆M13) (Fig. 1 and Table S2).

Despite of repeated attempts, we failed to enforce proper deletions of ∆M06–M09 (Fig. 1, dashed bars), which all include the region R2, thereby supporting the assumption by Lohße et al. (2011) of a non-deletable part in this region. By deletions ∆M14 and ∆M15 this non-deletable part was narrowed down to a region of 15.2 kb including *msr1_02770*–*msr1_03000* (Fig. 1), which in addition to several hypothetical genes encodes a putative toxin-antitoxin system (*msr1_02860*–*msr1_02870*) that might prevent its simultaneous deletion.

For all other targets, mutants could be readily generated as intended, yielding strains ∆M01–∆M05 and ∆M10–∆M17 with defined single deletions ranging from ~ 2.5 kb (*feoAB1op*, deleted in a later step) up to ~ 100 kb (∆M13) (Fig. 1, grey bars). The ∆A13 mutant from Lohße et al. (2011) (not to be confused with ∆M13, this study), already lacking *mms6op*, *mamGFDCop* and *mamXYop* (Fig. 1), was used as parental strain for the additional deletion of *mamABop* and *feoAB1op* to generate ∆M01 and ∆M03 mutants, respectively. To generate ∆M02, strain ∆A13∆*mms5*/*mmxF* lacking *mms6op*, *mamGFDCop*, *mamXYop* and *mms5*/*mmxF* (R. Uebe, unpublished) was used to delete the *mamABop*. Further deletion of regions R4 and R6 in the ∆M02 background then yielded ∆M05 (Fig. 1). All other deletions were introduced into the WT parent. ∆M01–∆M05 showed WT-like cell size, shape and morphology, but displayed slightly impaired swimming motility as their parent strains ([29], R. Uebe, unpublished).

**Fig. 3:**
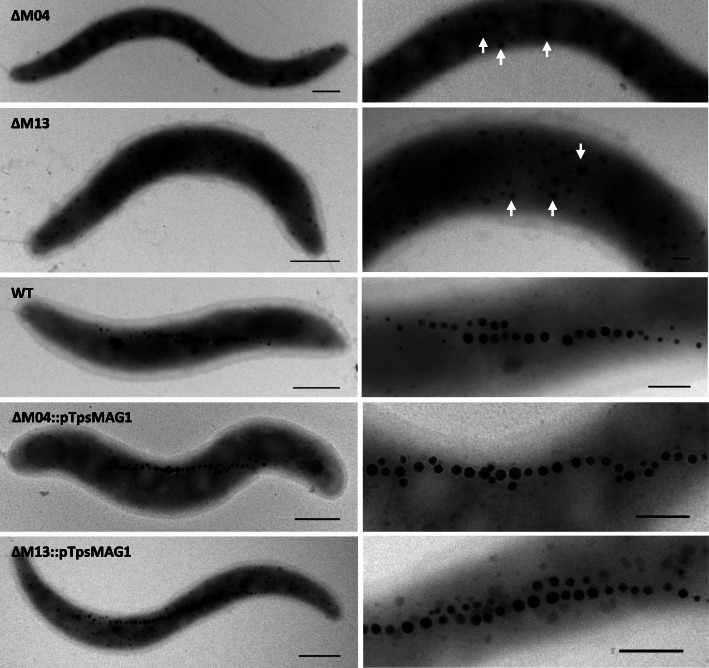
Phenotypes of non-magnetic mutant strains with largest deletion extents and their respective complemented strains with restored magnetosome biosynthesis. Mutants ∆M04 and ∆M13 are non-magnetic, while complemented mutants show WT-like magnetosome formation. Arrows indicate electron dense particles (EDPs) in mutant strains. Scale bars: left column, 500 nm; right column, 100 nm

As expected, all deletions comprising the known magnetosome clusters were impaired in magnetosome biosynthesis to different degrees. Mutants ∆M01–∆M05 and ∆M12–∆M13 lacking the *mamABop* were entirely devoid of magnetosomes, whereas ∆M11 (deletion of R7 with *mamXYop*, but all other *mam*/*mms/feo* clusters still present) essentially phenocopied the known intermediate magnetic phenotype typically caused by mutation of the *mamXYop* (Figs. 3 and S1) [36]. This phenotype is characterized by a reduced (40–80% of the WT) *C*_*mag*_ (a light-scattering based proxy for the average magnetic orientation of bacterial cells in liquid media [37]), with WT-like magnetite crystals flanked within the magnetosome chain by poorly crystalline flake-like particles. By contrast, elimination of regions outside the *mam/mms/feo* clusters (∆M10, ∆M14–∆M17 in R2 and R8) resulted in a WT-like magnetosome phenotype (Fig. S1). These mutants ∆M10 and ∆M14–∆M17, covering 15 putative mobile genetic elements, phage-related genes and several hypothetical genes, also displayed a WT-like cell growth at 28 °C under aerobic conditions (data not shown).

**Fig. 4:**
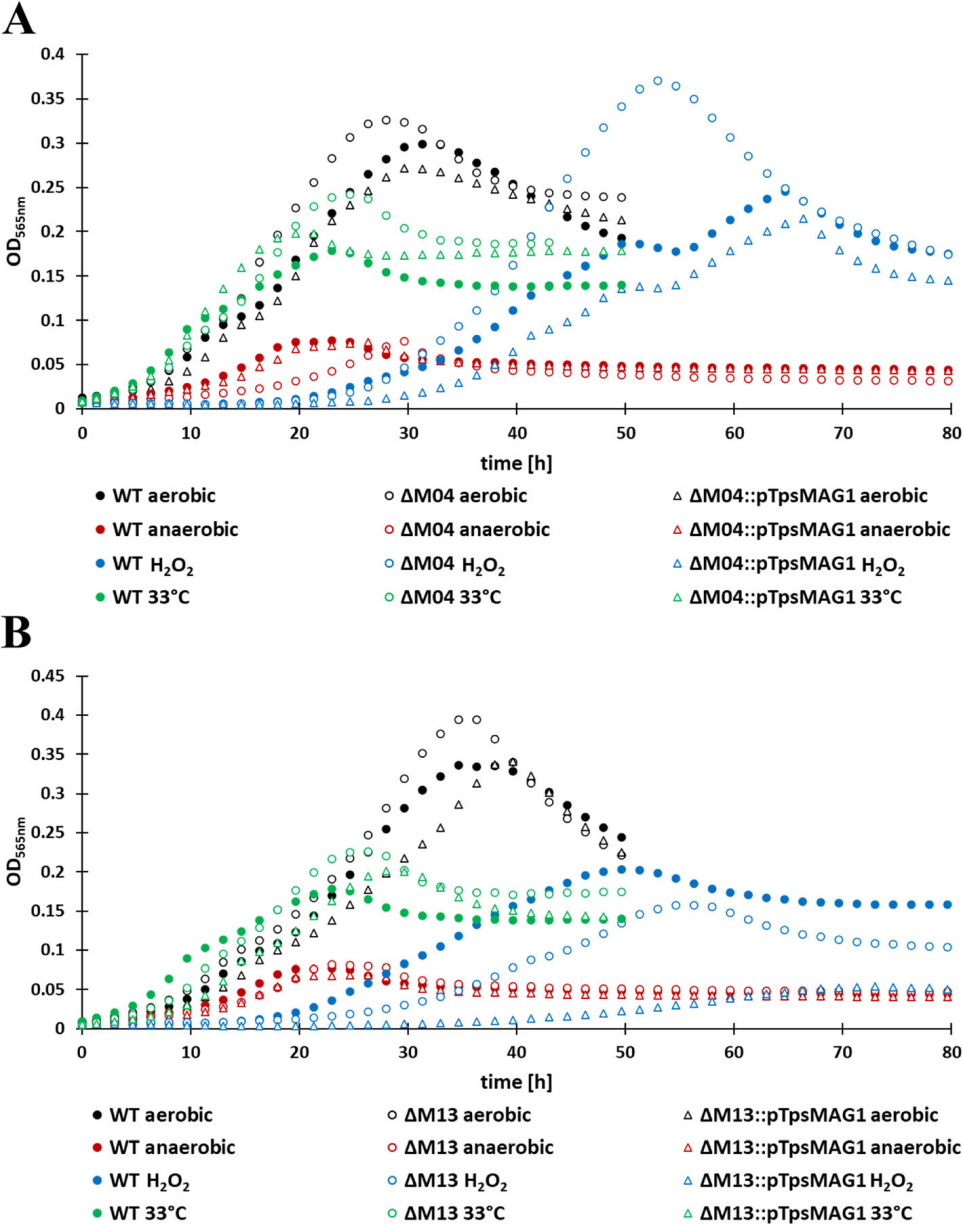
Growth characteristics of different mutants. Provided are the growth curves of non-magnetic mutant strains with largest deletion extents and their respective complemented strains with restored magnetosome biosynthesis. Growth curves show ∆M04 (**a**), ∆M13 (**b**) and its respective complemented mutants under different growth conditions in comparison to the WT. Growth of the WT is shown in diagrams for both mutants. Each strain was analyzed in technical triplicates, and growth curves represent the average while standard deviation was below 5%

However, all non-magnetic mutant strains in which deletions covered the *mamABop* (∆M01–∆M04) displayed a growth advantage over the WT by reaching higher cell densities (ca. 10–35%) under aerobic conditions or moderate heat stress at 33 °C (Fig. 4). An exception was strain ∆M05, which showed the same mild growth deficiency (lower cell yields) as its parent*,* probably due to an unidentified spontaneous second site mutation. Growth of non-magnetic ∆M01–∆M04 and ∆M13 mutants under anaerobic conditions was indistinguishable from the WT. However, in the presence of oxidative stress generated by H_2_O_2_, ∆M01–∆M04 grew to higher, and ∆M13 to lower densities than the WT, respectively (Fig. 4). Deleted genes in ∆M13 include a putative aerotaxis-related gene and several hypothetical genes, the loss of which might have caused the decreased sensitivity to oxidative stress.

Next, we tested whether the magnetic phenotypes could be restored by a compact version of all key magnetosome biosynthesis operons. To this end, a transposable cassette comprising *feoAB1op*, *mms6op*, *mamGFDCop*, *mamABop*, and *mamXYop* without intervening gene content was utilized. This cassette was harbored on pTpsMAG1 comprising the MycoMar (*tps*) transposase gene [38]. Reinsertion of the cassette at several random chromosomal locations in ∆M01–∆M04 and ∆M13 restored magnetosome biosynthesis to WT-levels (Figs. 3 and S1). This again confirmed that deleted genes apart from the *mam*/*mms* gene clusters are dispensable for magnetosome biosynthesis in *M. gryphiswaldense*. The presence of an extra copy of the endogenous *feoAB1op* seems to have no effect on magnetosome biomineralization, but it should be removed in future engineering steps to avoid unintended recombination events. After ‘re-magnetization’, growth rates of ∆M01::pTpsMAG1–∆M04::pTpsMAG1 and ∆M13::pTpsMAG1 were reduced to WT-levels under aerobic conditions and moderate heat stress. These findings indicate that magnetosome biosynthesis represents a significant burden that prevents cells from reaching higher cell yields observed in non-magnetic mutants. Under anaerobic conditions, complemented ∆M01::pTpsMAG1–∆M04::pTpsMAG1 and ∆M13::pTpsMAG1 strains showed WT-like cell yields. Under oxidative stress, complemented ∆M04::pTpsMAG1 revealed slight growth deficiencies (reduction by ~ 12% of WT OD), while the complemented ∆M13::pTpsMAG1 exhibited significantly reduced growth compared to the WT (reduction by ~ 70% of WT-level; Fig. 4).

**Fig. 5:**
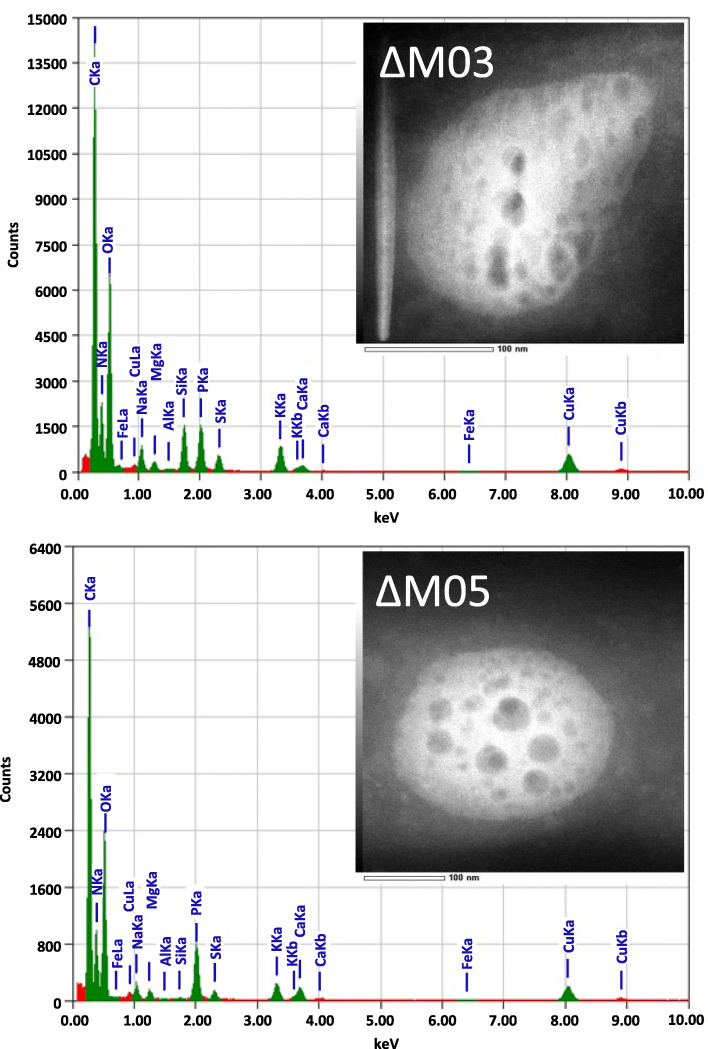
XEDS spectra and TEM micrographs (insets) of individual EDPs. EDPs were found in deletion strains ∆M01–∆M05 and ∆M13. Exemplary the mutants ∆M03 and ∆M05 are shown. Spectra indicate that EDPs are rich in potassium, phosphorus and oxygen, while no significant amounts of iron could be detected

Of note, in some of the non-magnetic mutants (∆M01–∆M05 and ∆M13) (Fig. 3) TEM revealed the presence of numerous (ca. 90 per cell) irregularly shaped conspicuous electron dense particles ranging 10–125 nm in size (in the following referred to as ‘EDP’), scattered over the entire cell. Analysis of strains ∆M03 and ∆M05 by high-resolution electron microscopy revealed that EDPs were amorphous. In addition, energy-dispersive X-ray spectroscopy (XEDS) showed that the inorganic inclusions were rich in potassium, phosphorus and oxygen, while no significant amounts of iron could be detected (Fig. 5). Variation of culture conditions such as growth in low-iron medium [25] supplemented with 10 μM 2,2′-dipyridyl as non-metabolizable iron chelator, or in medium oversaturated with 250 μM Fe (III)-citrate did not affect the number, size or appearance of EDPs (data not shown), confirming their independence from iron. Formation of EDPs was neither affected by variation of the phosphate concentration in the medium (0–3 mM), suggesting that low residual phosphate was still saturating for EDP formation. Furthermore, EDPs remained present in cells even after restoration of magnetosome biosynthesis by pTpsMAG1 complementation (Figs. 3 and S1). This indicates that the formation of EDPs is independent of magnetosome biosynthesis, but somehow linked to the deleted genes outside the five key magnetosome biosynthetic clusters. Because of their apparent irrelevance for magnetosome biosynthesis and growth, the identity and formation of EDPs was not explored further in this study.

Overall, the strain with the largest deletion that exhibited WT-like magnetosome biosynthesis upon complementation was ∆M13. In this mutant, a contiguous stretch of ~ 100 kb including all *mam* and *mms6* operons (~ 27 kb) but *feoAB1op*, interspaced or flanked by ~ 73 kb of irrelevant or problematic gene content was deleted and substituted by a contiguous, yet functional version of magnetosome biosynthetic gene clusters (Fig. 1).

### Deletion of putative determinants for magnetosome biosynthesis outside the MAI

**Fig. 6:**
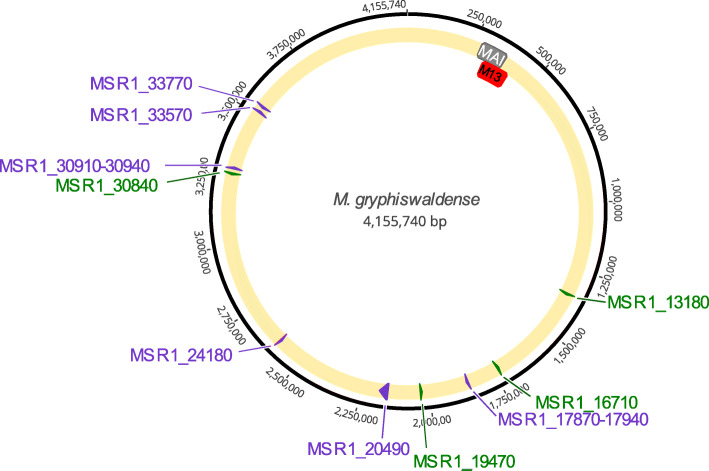
Schematic overview over the chromosomal positions of single deletions in this study. The yellow circle shows genes or gene sets targeted for deletion. Grey: MAI; red: M13 deletion; green: putative candidate genes for magnetosome biosynthesis outside the MAI [31, 32]

Next, we assessed the role of candidate genes with putative roles during magnetosome biosynthesis located outside the MAI. One group of these candidates was recently retrieved by genome-wide transposon mutagenesis, in which a colony appearance deviant from the dark-brown color of the WT served as a proxy for impaired magnetosome biomineralization [31]. Another category was comprised of candidate genes, whose gene products were found to be genuinely associated with magnetosome particles purified from disrupted *M. gryphiswaldense* cells [32]. Most interesting targets for mutagenesis were further selected based on their conservation in other magnetospirilla and/or a conspicuous genomic neighborhood. This resulted in the following list of deletion targets (Fig. 6; Table S3):

#### Candidates identified by Tn5-mutagenesis [31]

– A clone with a reduced *C*_*mag*_ was linked to a hit in *msr1_17870*, which is part of a putative operon comprising eleven genes (*msr1_17870*–*17940*) that is conserved in two other magnetospirilla (Table S3). It has predicted functions related to the TonB-system, which is known to form energized, gated pores that bind and internalize iron chelates in Gram-negative bacteria [39]. Here, we deleted the entire 9.5 kb operon region.

– Several Tn-insertants within a huge (31 kb) monocistronic gene (*msr1_20490*) became suspicious because of their slightly altered colony appearance [31]. The gene encodes a single giant putative surface protein with a predicted mass of 1147 kDa and a repetitive structure, which belongs to the FecR/concanavalin A-like lectin/glucanase superfamily [31]. It is also conserved in several other magnetic and non-magnetic magnetospirilla (Table S3). In our study, we deleted the entire open reading frame of *msr1_20490*.

– Conspicuously, *msr1_24180* was also hit by several independent Tn5-insertions [31] and is conserved in most magnetospirilla (Table S3). It contains a lysylphosphatidylglycerol synthase transmembrane region with putative function in cell wall modification [31]. We deleted *msr1*_*24180* (~ 1 kb) in this study.

– The first four genes (*msr1_30910*–*30940*) of a six-gene operon were hit several times independently [31] and are conserved in several magnetospirilla (Table S3). The predicted functions (e.g., a glycosyl transferase gene, a dTDP-sugar isomerase, a methyltransferase and epimerase/dehydratase (NAD) gene) may play an important role in cell wall biogenesis or modification reported by Silva et al. (2020). *msr1*_*30910*–*30940* (~ 3.5 kb) were deleted in this study.

– *msr1_33570* and *msr1_33770* are hypothetical genes which were also retrieved by the Tn-screen. They are conserved in many magnetospirilla (Table S3). Both genes were deleted (∆*msr1_33570*, 1.2 kb, ∆*msr1_33770*, 0.3 kb).

#### Candidates identified by magnetosome membrane proteomics [32]

- MSR1_13180 (10 kDa), MSR1_16710 (9 kDa) and MSR1_19470 (11 kDa) are transmembrane proteins with unknown functions, but orthologs in many magnetospirilla. All three respective genes were deleted individually (0.27 kb, 0.249 kb, 0.33 kb, respectively).
- MSR1_30840 is a transmembrane protein (33 kDa, four TMH) predicted as a putative peptidase, encoded next to potential LPS core biosynthesis genes, which is also conserved in two other magnetospirilla (Table S3). In addition to its detection in the magnetosome membrane [32], *msr1_30840* is within close genomic neighborhood (7.4 kb) to *msr1_30910*–*30940*, all having received several Tn5-hits [31]. ∆*msr1_30840* was generated in this study (0.951 kb).

Deletion mutants of all targeted genes could be obtained in a straightforward manner. Some of the null mutants (∆*msr1_20490*, ∆*msr1_30910–30940*, ∆*msr1_30840*) displayed a slightly reduced C_*mag*_ (< 1), compared to WT-levels of 1–2, and the cell shape of ∆*msr1_20490* seemed to be more spiralized. However, TEM analysis revealed the presence of magnetosomes apparently indistinguishable from the WT with respect to number, size, shape and alignment in all mutants (Fig. S2). Hence, contrary to the previous hypotheses, these genes play no obvious and strong role in magnetosome biosynthesis under the tested conditions.

## Discussion

In this study, we tested an approach for large-scale gene deletion in *M. gryphiswaldense* and employed it for the mutational analysis of candidate genes and the elimination of regions irrelevant for magnetosome biosynthesis. We extended the tested range of contiguous MAI deletions by ca. 13 kb compared to Lohße et al. (2011), and show that deletions of up to ~ 100 kb are feasible using allelic replacement based on homologous recombination with reasonable efficiency and time requirement. In total, we generated 24 deletions, ranging from about 0.25–100 kb in size and covering about 167.2 kb. However, we also revealed several pitfalls and potential caveats. When attempting to delete ‘recalcitrant’ or essential targets, false positive clones may arise, in which the second double-crossover had failed. Instead parts of the vector were retained in the genome through insertion by single homologous or non-homologous recombination, which was often associated with extensive spontaneous rearrangements of the adjacent regions. In all analyzed cases this was caused in the first place by spontaneous inactivation of the suicide gene *galK* by insertion of IS elements, which prohibited counterselection in the presence of galactose. This emphasizes the need of caution by sequence verification of the intended excision site.

Except for ∆M05, *mamABop* deficient non-magnetic deletants showed a growth advantage, which became lost upon ‘re-magnetization’ by complementation. Not surprisingly, magnetosome biosynthesis seems to impose a substantial metabolic burden, resulting in slower growth and lower yields compared to non-magnetic mutants. Neither the deletion of the MAI flanking regions nor any of the candidate genes outside the MAI had a strong and obvious effect on magnetosome biosynthesis, at least under the tested standard conditions. While this finding is unsurprising for the flanking regions, it may hint at an issue for the candidates retrieved in a recent Tn5-mutagenesis study. In these cases, the unaffected magnetosome phenotype of our clean gene deletions indicates that the observed reduced *C*_*mag*_ value or the deviant colony appearance of the Tn5-insertants [31] is likely due to subtle differences in cell shape and/or cell surface, rather than to direct effects on magnetosome biosynthesis. This is consistent with the functional prediction of several of these genes in pathways related to cell envelope biosynthesis. However, candidates identified by a previous proteomic study as constituents of the magnetosome membrane are unlikely to simply represent false positives due to contaminations because of the rigorous magnetosome purification procedure [32]. Instead, these proteins are likely to be indeed native constituents of this compartment, but their function may be only required in conditions not tested in our study or can be substituted by other magnetosome proteins.

## Conclusion

Our results further delineate the genetic complement for magnetosome biosynthesis. We engineered a strain, in which a ~ 100 kb region comprising large parts of the MAI and flanking regions was substituted by a compact (~ 38 kb), yet fully functional cassette containing the five key magnetosome biosynthetic operons *mamGFDCop*, *mms6op*, *mamABop*, *mamXYop*, and *feoAB1op*, but devoid of any flanking and intervening regions. The elimination of about 73 kb of genetic junk and 39 putative mobile genetic elements (equivalent to ~ 33% of all known putative mobile genetic elements in the genome of *M. gryphiswaldense*) may contribute to increased genetic stability, as already suggested by a recent study [40].

## Methods

### Bacterial strains, vectors, and cultivation conditions

Bacterial strains and plasmids used in this study are listed in Table S1. *Escherichia coli* strains were grown as previously described [41]. For the cultivation of *E. coli* WM3064 lysogeny broth (LB) medium was supplemented with 25 μg/ml (final concentration) kanamycin (Km), 15 μg/ml gentamycin (Gm), 12 μg/ml tetracycline (Tet) and 1 mM DL-α,ε-diaminopimelic acid (DAP). Liquid cultures of *M. gryphiswaldense* strains were grown microaerobically in flask standard medium (FSM) [5] at 28 °C under moderate shaking (120 rpm), and strains carrying the suicide or the Cre plasmids were cultivated by adding 5 μg/ml Km, 20 μg/ml Gm or 5 μg/ml Tet. For cultivation on solid LB medium and FSM, 1.5% (w/v) agar was added. Cultivation from single *M. gryphiswaldense* colonies was performed by transferring cell material into 150 μl FSM in 96-deep-well-plates (Eppendorf, Hamburg, Germany), prior to gradually increasing the culture volume. The optical density (OD) at 565 nm and magnetic response (*C*_*mag*_, i.e., a proxy for the average magnetic orientation of bacterial cells in liquid media based on light-scattering) of cells in the exponential growth phase were measured photometrically as previously reported [37].

Growth experiments were performed by using pre-cultures grown for two daily passages under microaerobic conditions at 28 °C. Cultures were adjusted to an initial OD of 0.01 and grown in an Infinite F200pro microplate reader (Tecan, Switzerland) under aerobic conditions at 28 °C or moderate heat stress at 33 °C. For induction of oxidative stress, 20 μM H_2_O_2_ were added prior to starting the growth experiments.

### Molecular and genetic techniques

Oligonucleotides used as primers for amplification of DNA fragments were deduced from the working draft genome sequence of *M. gryphiswaldense* (GenBank accession number CP027526) [42] and purchased from Sigma-Aldrich (Steinheim, Germany). Plasmids were constructed by standard recombinant techniques as described below. Generated constructs were sequenced by Macrogen Europe (Amsterdam, Netherlands) and sequence data analyzed with Geneious 8.0.5 (Biomatters Ltd., New Zealand).

#### Construction of loxP site vectors and mutant strains

Upstream and downstream regions of about 1–2.5 kb of deletion targets were amplified and subcloned into *loxP* suicide plasmids pAL01 and pAL02/2 [29], respectively. Resulting vectors were sequence-verified by PCR and conjugated into *M. gryphiswaldense* using *E. coli* WM3064 as donor strains. Insertion mutants were distinguished from the WT by Km, and Km plus Gm selection. Addition of *Cre* recombinase plasmid pLYJ87 [43] by conjugational transfer resulted in the excision of target regions, and the plasmid was subsequently cured from each mutant by several transfers in FSM without any antibiotics. Deletions were verified by PCR and sequencing.

#### Construction of markerless gene deletion vectors and mutants

Generation of single and multiple deletion mutants was accomplished by a tailored *galK* counterselection system as described previously [34] (Fig. S1). The pORFM-GalK-vector was digested using EcoRV to insert fused upstream and downstream fragments each of about 1–2.5 kb. For larger fragments (> 20 kb), flanking regions between 1.5–2.5 kb were amplified while for deletion of smaller fragments, homologous regions < 1.5 kb were used. Proper construction of resulting plasmids was verified by PCR and sequencing. The latter were transferred into *M. gryphiswaldense* strains by conjugation using *E. coli* WM3064 as donor. Genomic insertion mutants were identified using a kanamycin resistance marker (Km^r^, aminoglycoside 3′-phosphotransferase type IIa encoded by the *aph(3′)-IIa* gene) [44] which was present on the suicide vector. After ~ 5 d of incubation at 28 °C, Km^r^ clones were picked and re-grown in up to 1 ml FSM at 28 °C. For generation of double crossover mutants, selected clones were plated onto FSM agar containing 2.5% galactose to counterselect for vector integration by the lethal activity of galactokinase (GalK). This enzyme catalyzes the phosphorylation of galactose. Since *M. gryphiswaldense* is unable to metabolize galactosephosphate, this product accumulates to toxic levels inside the cell. As a result, only cells that have undergone a second recombination event and thus, have removed the plasmid backbone, are able to survive. Deletions were verified by PCR and sequencing.

### Analytical methods

#### Re-sequencing of genomic DNA

Genomic DNA (gDNA) was isolated following the manual instructions of Quick-DNA Midiprep Plus Kit (Zymo Research Europe GmbH). For each isolated gDNA, two sequencing libraries were prepared, one for sequencing on the MiSeq platform (Illumina Inc., NL), and one for sequencing on the GridION platform (Oxford Nanopore Technologies (ONT), UK). The former was constructed using the TruSeq DNA PCR-free Library Kit (Illumina Inc., The Netherlands) and was run in a 2 × 300 nt run using a 600 cycle MiSeq Reagent Kit v3 (Illumina Inc., The Netherlands). For ONT sequencing, the Ligation Sequencing Kit SQK-LSK109 was used to prepare the libraries, which were in turn run on a R9.4.1 flow cell. Basecalling of the raw ONT data was performed with guppy v3.2.8 [45]. For assembly, three assemblers were utilized: The canu assembler v1.8 [46] was used to assemble the ONT data. The resulting assembled contigs were polished using first the ONT data with racon v1.3.3 [47] and medaka v0.11.5 (Oxford Nanopore Technologies), both relying on minimap2 v2.17-r943 [48] for mapping, followed by switching to the Illumina data and the pilon polisher v1.22 [49] for a total of 10 rounds. For the first 5 rounds, bwa mem [50] was used as a mapper, for the final 5 cycles, bowtie2 [51] was applied. In addition, the Illumina data was assembled using newbler v2.8 [52] and both data sets were assembled using unicycler [53]. All assemblies were compared with each other and checked for synteny using r2cat [54]. All three assemblies were combined and manually curated using consed [55]. Annotation of the finished genomes was performed using prokka v1.11 [56] SNPs and small indels were identified using snippy v4.0 [57] while larger rearrangements were identified manually using SnapGene (GSL Biotech).

#### Preparation of samples for transmission electron microscopy (TEM)

For routine TEM of cell and magnetosome morphologies, cultures were grown under microoxic conditions in FSM. Overnight cultures were fixed in 1.5% formaldehyde and deposited onto carbon-coated copper-mesh grids (Science Services, Munich, Germany). TEM was performed on a JEOL 1400 (Japan) with an acceleration voltage of 80 kV. Micrographs were analyzed using the software ImageJ [58].

For analysis of unidentified electron dense particles (uEDP), bright field TEM, high-resolution (HR) TEM and energy-dispersive X-ray spectroscopy (XEDS) were performed on a spherical aberration corrected JEOL ARM 2100 at an acceleration voltage of 200 kV and an emission current of 10 μA.

## Supplementary Information

**Additional file 1.**

## Data Availability

The datasets used and analyzed during the current study are available from the corresponding author on reasonable request. Raw sequencing data are available under BioProject number PRJNA691753.

## Abbreviations

MAI: Magnetosome Island
WT: Wild type
IS element: Insertion element
*tn-tandem*: Transposon-tandem
Km^r^: Kanamycin resistant
EDP: Electron dense particles
DAP: DL-α,ε-diaminopimelic acid.
