# Supplementary material for: Identification and elimination of genomic regions irrelevant for magnetosome biosynthesis by large-scale deletion in Magnetospirillum gryphiswaldense
Source: BMC Microbiol. 2021 Feb 25;21:65. doi: 10.1186/s12866-021-02124-2 (PMC7908775; doi:10.1186/s12866-021-02124-2)
Supplement: Supplementary file 1 — Additional file 1. [file 12866_2021_2124_MOESM1_ESM.pdf]

Supplementary Material

Identification and elimination of genomic regions irrelevant for magnetosome biosynthesis by large-scale deletion in *Magnetospirillum gryphiswaldense*

Theresa Zwiener[1], Frank Mickoleit[1], Marina Dziuba[1,2], Christian Rückert[3], Tobias Busche[3], Jörn Kalinowski[3], Damien Faivre[4,5], René Uebe[1], and Dirk Schüler[1#]

[1]Department of Microbiology, University of Bayreuth, Bayreuth, Germany

[2]Institute of Bioengineering, Research Center of Biotechnology of the Russian Academy of Sciences, Moscow, Russia

[3]Center for Biotechnology, University of Bielefeld, Bielefeld, Germany

[4]Max Planck Institute of Colloids and Interfaces, Department of Biomaterials, Potsdam, Germany

[5]Aix-Marseille Université, CEA, CNRS, BIAM 13108, Saint Paul lez Durance, France

#Address correspondence to Dirk Schüler, dirk.schueler@uni-bayreuth.de.

Table S1. Bacterial strains and plasmids used in this study.

Bacterial strain or plasmid	Relevant characteristics	Reference
**Strains**		
E. coli		
DH5α	F⁻ *sup*E44 Δ*lac*U169 (Φ80*lac*ZDM15) *hsd*R17 *rec*A1 *end*A1 *gyr*A96 *thi*-1 *rel*A1	Invitrogen
WM3064	*thrB1004 pro thi rpsL hsdS lacZΔM15 RP4-1360 Δ(araBAD)567ΔdapA1341::[erm pir]*	W. Metcalf (unpublished)
M. gryphiswaldense	Archetype	(1)
M. gryphiswaldense ΔA13	R3/S1, Δ*mms6*/*mamGFDCop*, Δ*mamXY*	(2)
M. gryphiswaldense ΔA13Δ*mms5*/*mmxF*	R3/S1, Δ*mms6*/*mamGFDCop*, Δ*mamXY*, Δ*mms5*/*mmxF*	R. Uebe (unpublished)
**Plasmids**		
pTZ_028	pAL01 with *lox71*, insertion of homologous sequence *mamABop*	This study
pTZ_029	pAL02/2 with *lox66*, insertion of homologous sequence *mamABop*	This study
pTZ_030	pAL01 with *lox71*, insertion of homologous sequence M04	This study
pTZ_031	pAL02/2 with *lox66*, insertion of homologous sequence M04	This study
pTZ_052	pORFM-GalK derivate, insertion of homologous sequence MSR1_17870–17940	This study
pTZ_053	pORFM-GalK derivate, insertion of homologous sequence MSR1_19470	This study
pTZ_055	pORFM-GalK derivate, insertion of homologous sequence MSR1_13180	This study
pTZ_057	pORFM-GalK derivate, insertion of homologous sequence MSR1_30910–30940	This study
pTZ_058	pORFM-GalK derivate, insertion of homologous sequence MSR1_33570	This study

pTZ_057	pORFM-GalK derivate, insertion of homologous sequence MSR1_20490	This study
pTZ_061	pORFM-GalK derivate, insertion of homologous sequence *mamABop*	This study
pTZ_067	pORFM-GalK derivate, insertion of homologous sequence M04	This study
pTZ_070	pORFM-GalK derivate, insertion of homologous sequence MSR1_16710	This study
pTZ_071	pORFM-GalK derivate, insertion of homologous sequence MSR1_30840	This study
pTZ_083	pORFM-GalK derivate, insertion of homologous sequence MSR1_33770	This study
pTZ_088	pORFM-GalK derivate, insertion of homologous sequence M06	This study
pTZ_089	pORFM-GalK derivate, insertion of homologous sequence M10	This study
pTZ_092	pORFM-GalK derivate, insertion of homologous sequence *intA1*	This study
pTZ_093	pORFM-GalK derivate, insertion of homologous sequence *intA2*	This study
pTZ_094	pORFM-GalK derivate, insertion of homologous sequence P1.3	This study
pTZ_096	pORFM-GalK derivate, insertion of homologous sequence M15	This study
pTZ_097	pORFM-GalK derivate, insertion of homologous sequence M16	This study
pTZ_102	pORFM-GalK derivate, insertion of homologous sequence M07	This study
pTZ_103	pORFM-GalK derivate, insertion of homologous sequence M08	This study
pTZ_104	pORFM-GalK derivate, insertion of homologous sequence M11	This study
pTZ_105	pORFM-GalK derivate, insertion of homologous sequence M12	This study

pTZ_106	pORFM-GalK derivate, insertion of homologous sequence M14	This study
pTZ_107	pORFM-GalK derivate, insertion of homologous sequence M17	This study
pTZ_114	pORFM-GalK derivate, insertion of homologous sequence M13	This study
pLYJ87	Tet^r, *Cre* recombinase plasmid	(3)
pTpsMAG1	Km^r, Cm^r, p15A ori, mariner tps, *mamAB*, *mamGFDC*, *mms6*, *mamXY*, *feoAB1*	(4)

Table S2. Overview over single deletion mutants of MAI and its adjacent region.

**Name of single deletion mutant**	**Deleted genes**	**Total extent of deletion**	**Description**
**ΔM01**	MSR1_03150 to MSR1_03220	27,424 bp	Non-magnetic, electron-dense particles
	MSR1_03340 to MSR1_03500		
	MSR1_03850 to MSR1_03880		
**ΔM02**	MSR1_02690 to MSR1_02700	28,419 bp	Non-magnetic, electron-dense particles
	MSR1_03150 to MSR1_03220		
	MSR1_03340 to MSR1_03500		
	MSR1_03850 to MSR1_03880		
**ΔM03**	MSR1_02660 to MSR1_02670	29,830 bp	Non-magnetic, electron-dense particles
	MSR1_03150 to MSR1_03220		
	MSR1_03340 to MSR1_03500		

	MSR1_03850 to MSR1_03880		
**ΔM04**	MSR1_03150 to MSR1_03880	65,965 bp	Non-magnetic, electron-dense particles
**ΔM05**	MSR1_02690 to MSR1_02700	66,959 bp	Non-magnetic, electron-dense particles
	MSR1_03150 to MSR1_03880		
**ΔM06**	MSR1_02660 to MSR1_03140	35,049 bp	Not deletable in this study
**ΔM07**	MSR1_02660 to MSR1_03330	51,271 bp	Not deletable in this study
**ΔM08**	MSR1_02660 to MSR1_03500	67,533 bp	Not deletable in this study
**ΔM09**	MSR1_02660 to MSR1_03880	100,738 bp	Not deletable in this study
**ΔM10**	MSR1_03890 to MSR1_04210	32,801 bp	WT-like magnetic phenotype
**ΔM11**	MSR1_03510 to MSR1_04210	66,204 bp	WT-like magnetite crystals flanked by flake-like particles (5)
**ΔM12**	MSR1_03340 to MSR1_04210	82,756 bp	Non-magnetic, electron-dense particles
**ΔM13**	MSR1_03150 to MSR1_04210	98,984 bp	Non-magnetic, electron-dense particles
**ΔM14**	MSR1_03010 to MSR1_03140	11,238 bp	WT-like magnetic phenotype
**ΔM15**	MSR1_02680 to MSR1_02770	6,198 bp	WT-like magnetic phenotype
**ΔM16**	MSR1_03890 to MSR1_04010	10,849 bp	WT-like magnetic phenotype
**ΔM17**	MSR1_04020 to MSR1_04210	21,952 bp	WT-like magnetic phenotype
**Δf*eoAB1op***	MSR1_02660 to MSR1_02670	2,406 bp	weakly magnetic

Table S3. Overview of all single deletion mutants outside the MAI.

Name of single deletion mutant	Extent of deletion (bp)	Phenotype	Species with orthologs present
**ΔMSR1_17870–MSR1_17940**	9,504	WT-like C_{mag} and magnetosomes	*Magnetospirillum* sp. 64-120, *M. aberrantis*
**ΔMSR1_20490**	31,026	reduced C_{mag}, WT-like magnetosomes, more spiralized cell shape, conserved in	*M. moscoviense, Magnetospirillum* sp. LM-5, *M. marisnigri, Magnetospirillum* sp. 15-1, *Magnetospirillum* sp. UT-4, *Magnetospirillum* sp. ME-1
**ΔMSR1_24180**	1,008	WT-like C_{mag} and magnetosomes, conserved in	*Magnetospirillum* sp. 64-120, *Magnetospirillum* sp. UT-4, *Magnetospirillum* sp. LM-5, *M. moscoviense, M. aberrantis, Magnetospirillum* sp. 15-1, *M. marisnigri, M. kuznetsovii, Magnetospirillum* sp. SS-4, *M. magneticum, M. magnetotacticum, Magnetospirillum* sp. XM-1
**ΔMSR1_30910–MSR1_30940**	3,493	reduced C_{mag}, WT-like magnetosomes	*M. caucaseum, M. magnetotacticum, M. magneticum, Magnetospirillum* sp. XM-1, *Magnetospirillum* sp. SS-4, *Magnetospirillum* sp. ME-1, *M. marisnigri, Magnetospirillum* sp. 15-1
**ΔMSR1_33570**	1,233	WT-like C_{mag} and magnetosomes	*Magnetospirillum* sp. 64-120, *M. moscoviense, M. magnetotacticum, M. marisnigri, Magnetospirillum* sp. XM-1
**ΔMSR1_33770**	306	WT-like C_{mag} and magnetosomes	*Magnetospirillum* sp. 64-120, *M. moscoviense, M. magnetotacticum, M. marisnigri, Magnetospirillum* ME-1, *Magnetospirillum* sp. XM-1, *M. caucaseum*

**ΔMSR1_13180**	270	WT-like C_{mag} and magnetosomes	*Magnetospirillum* sp. 64-120, *Magnetospirillum* sp. XM-1, *M. magnetotacticum*, *M. caucaseum*, *M. moscoviense*, *M. magneticum*
**ΔMSR1_16710**	249	WT-like C_{mag} and magnetosomes	*Magnetospirillum* sp. 64-120, *Magnetospirillum* sp. LM-5, *M. moscoviense*, *Magnetospirillum* sp. UT-4, *M. aberrantis*, *M. marisnigri*, *Magnetospirillum* sp. SS-4, *M. caucaseum*, *Magnetovibrio blakemorei*, *M. magnetotacticum*, *M. kuznetsovii*
**ΔMSR1_19470**	330	WT-like C_{mag} and magnetosomes	*Magnetospirillum* sp. SS-4, *M. aberrantis*, *M. magneticum*, *M. kuznetsovii*. *M. caucaseum*, *Magnetospirillum* sp. ME-1, *Magnetospirillum* sp. XM-1, *Magnetospirillum* sp. 15-1, *Magnetospirillum* sp. UT-4
**ΔMSR1_30840**	951	reduced C_{mag}, WT-like magnetosomes	*Magnetospirillum* sp. 64-120, *M. moscoviense*

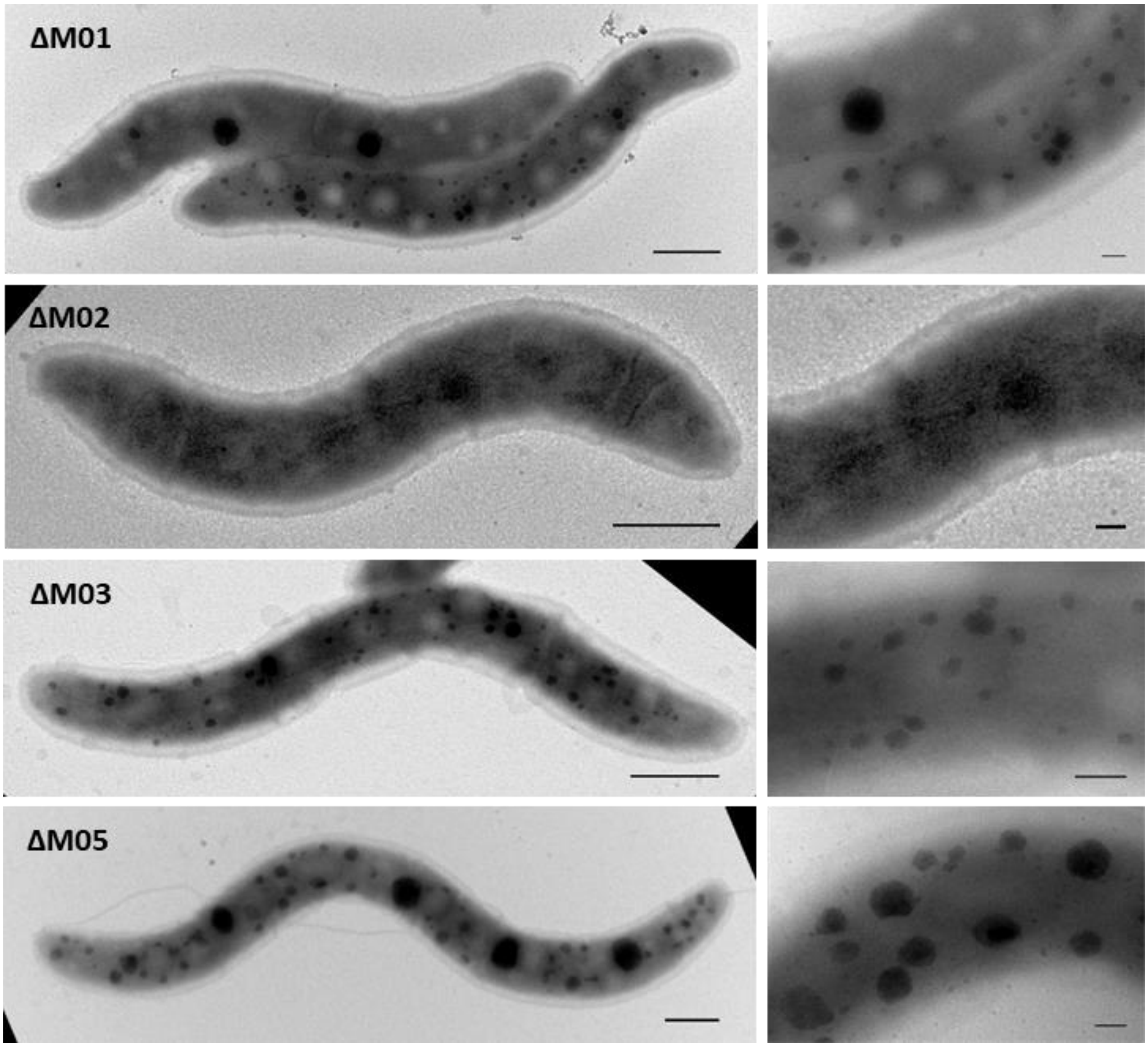
ΔM01
ΔM02
ΔM03
ΔM05

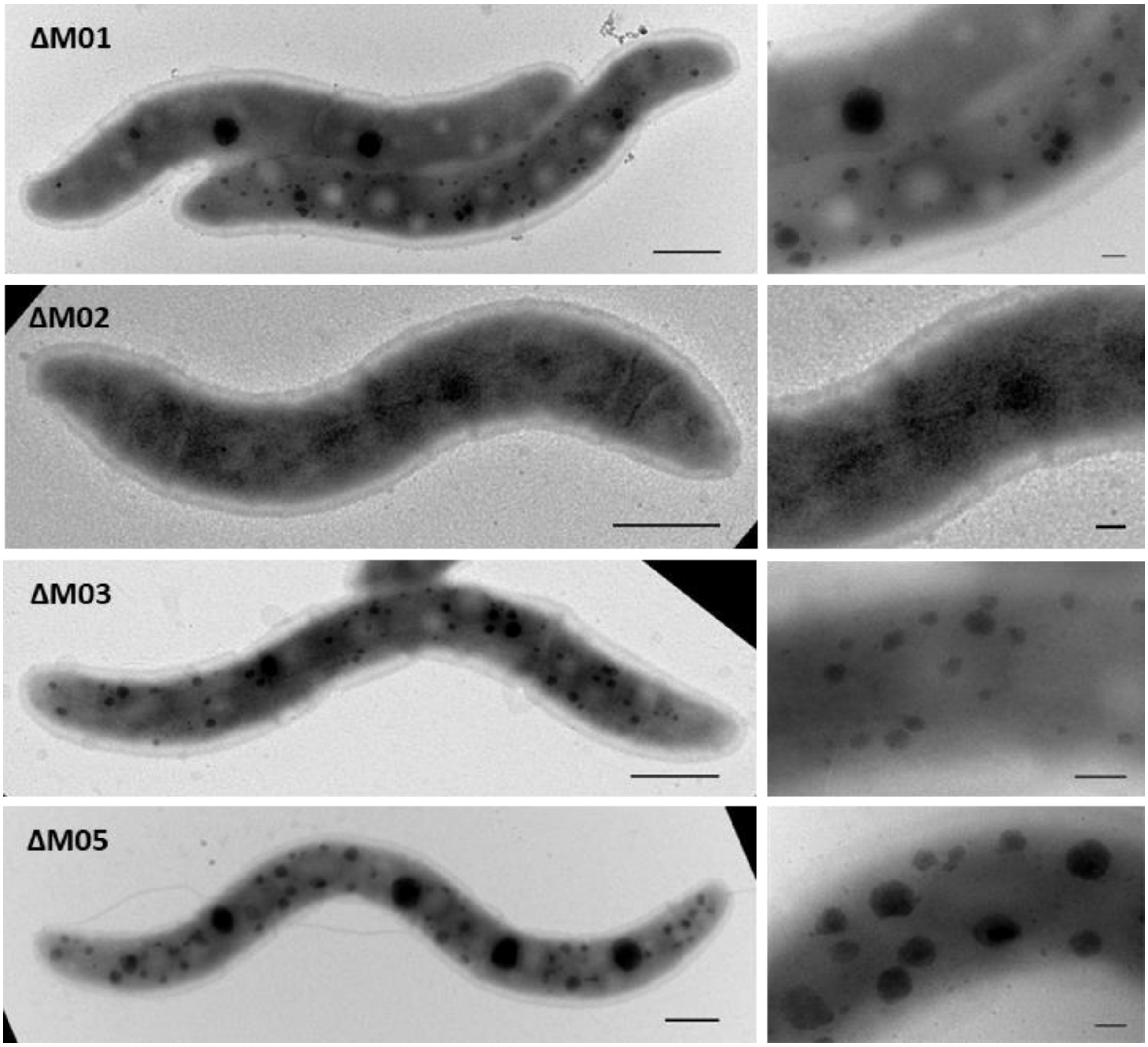
ΔM01
ΔM02
ΔM03
ΔM05

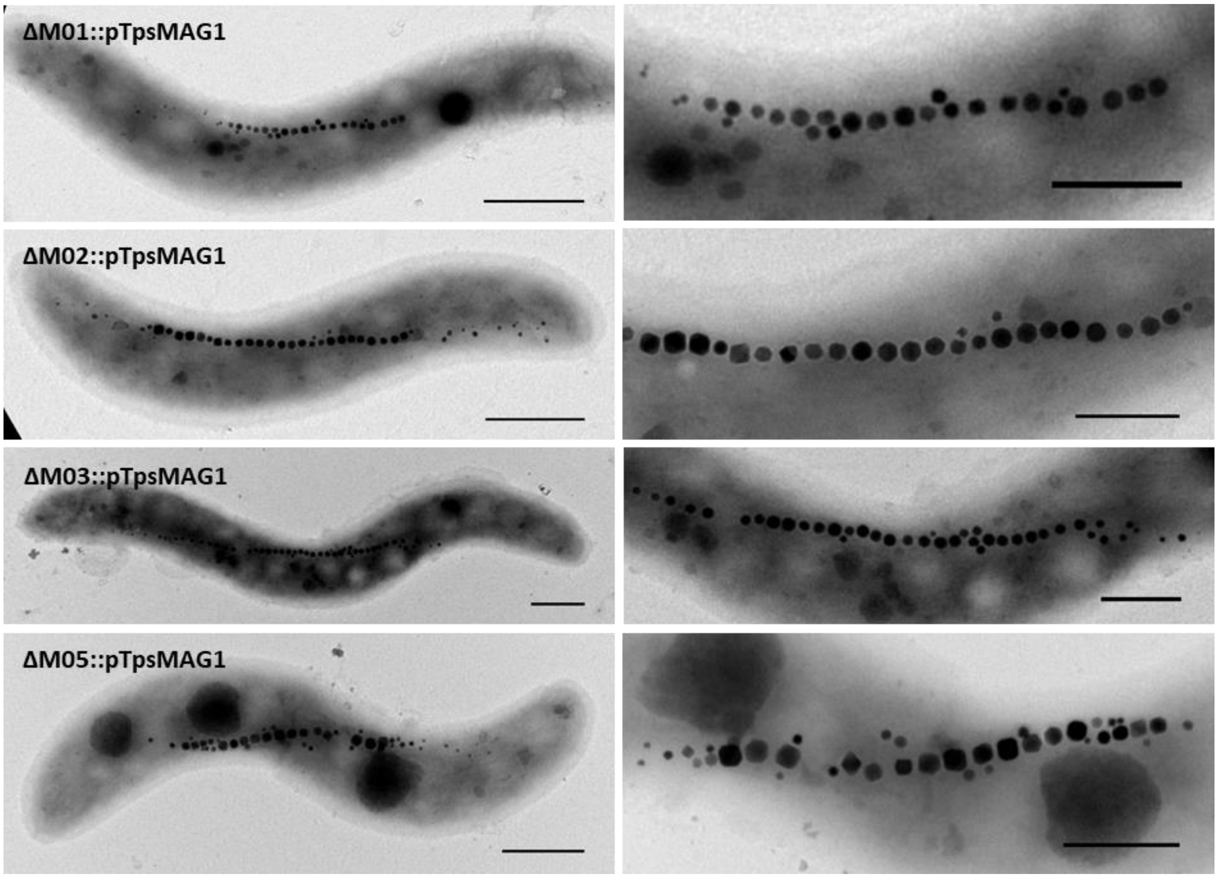
ΔM01::pTpsMAG1
ΔM02::pTpsMAG1
ΔM03::pTpsMAG1
ΔM05::pTpsMAG1

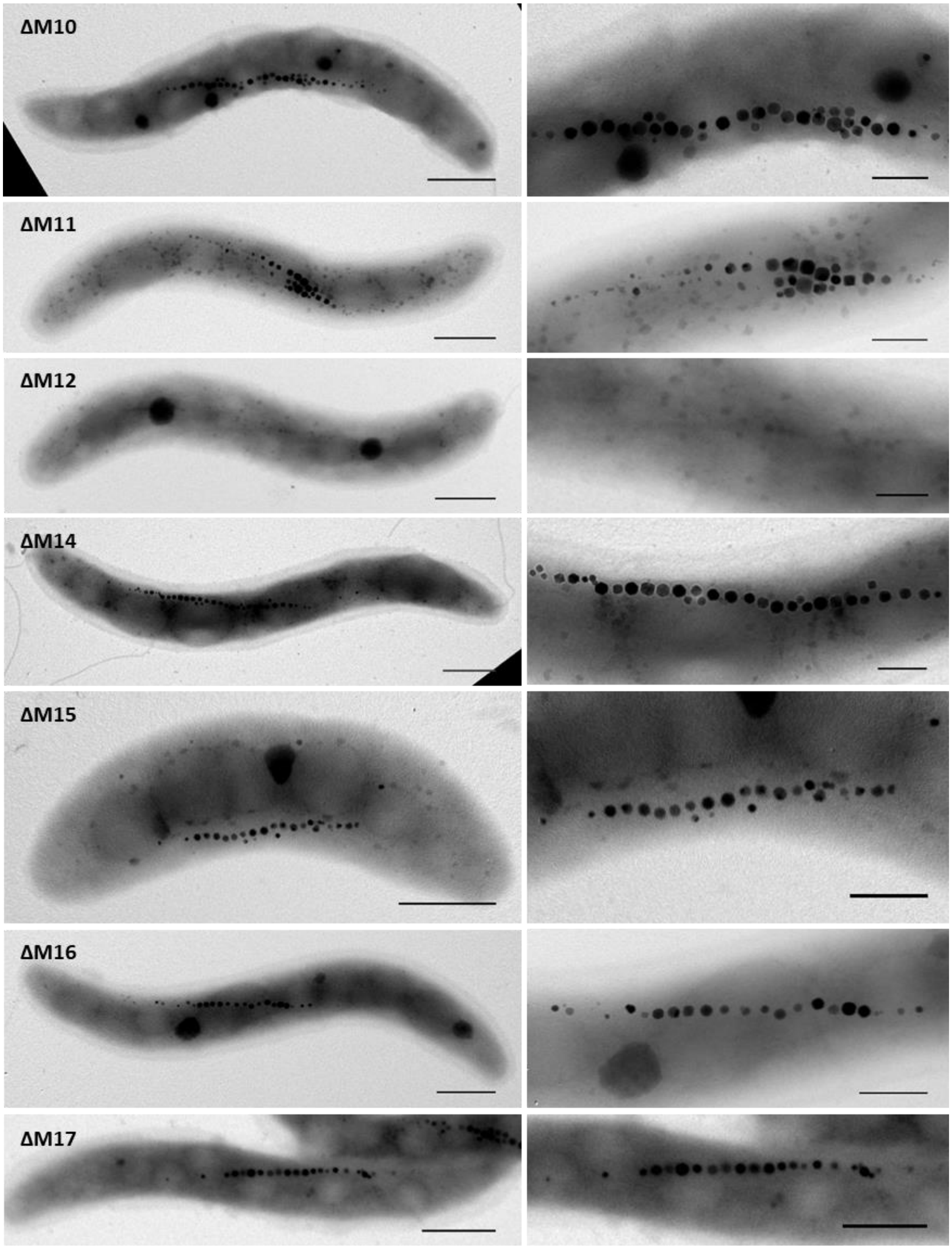

Figure S1. Morphology of all single deletion mutants of the MAI and adjacent regions and its complemented strains. Scale bars left side: 500 nm; right side: 100 nm.

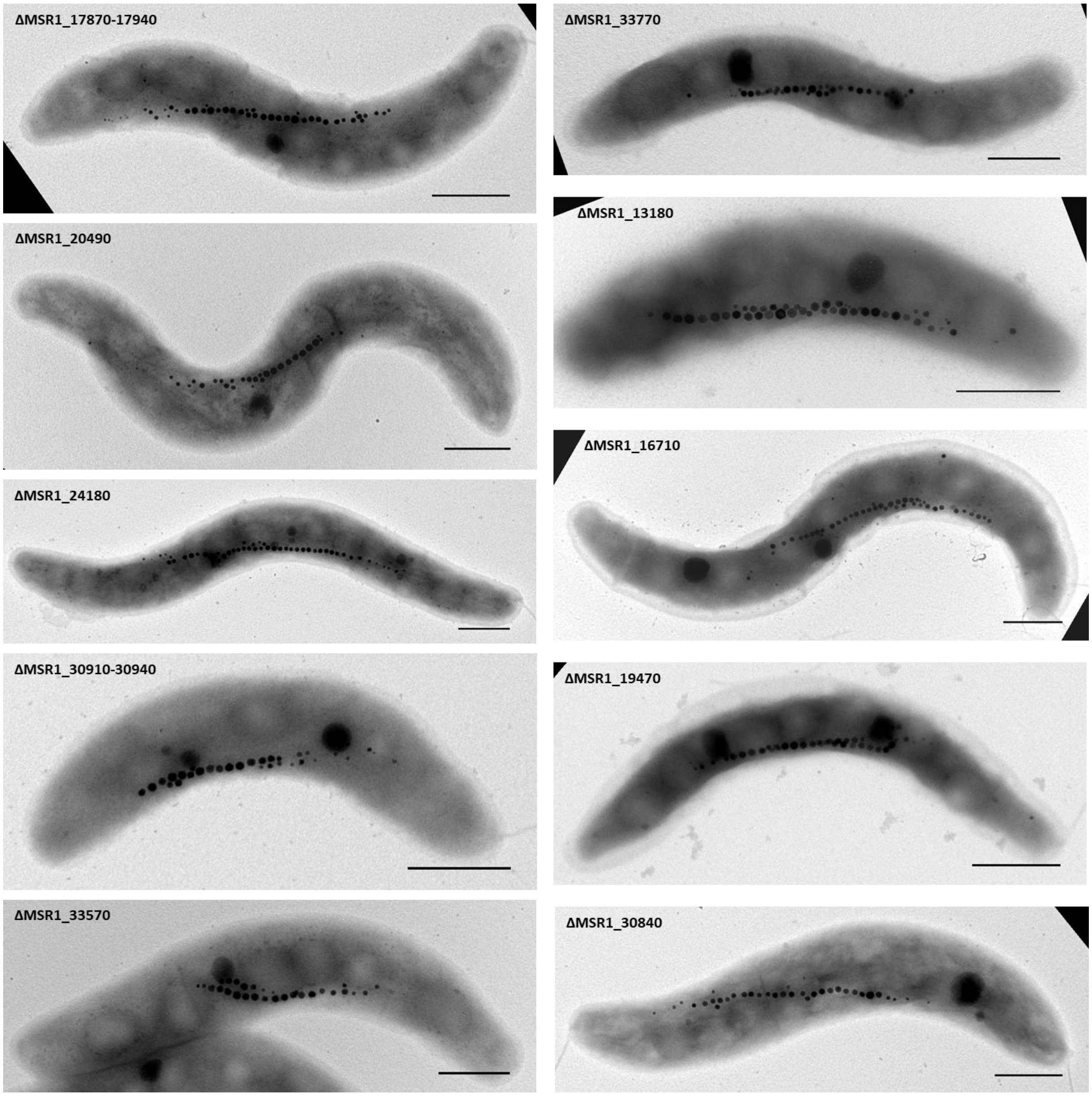

Figure S2. Single deletion mutants of Tn5-hits, candidates from proteome analysis and further gene clusters. Scale bars: 500 nm.

References Supplementary Material

1. Schüler D, Köhler M. The isolation of a new magnetic spirillum. Zentralblatt Mikrobiologie. 1992;147:150–51.
2. Lohße A, Ullrich S, Katzmann E, Borg S, Wanner G, Richter M, Voigt B, Schweder T, Schüler D. Functional analysis of the magnetosome island in *Magnetospirillum gryphiswaldense*: the *mamAB* operon is sufficient for magnetite biomineralization. PLoS One. 2011;6:e25561.
3. Li YJ, Bali S, Borg S, Katzmann E, Ferguson SJ, Schüler D. Cytochrome cd_1 nitrite reductase NirS is involved in anaerobic magnetite biomineralization in *Magnetospirillum gryphiswaldense* and requires NirN for proper d_1 heme assembly. J Bacteriol. 2013;195:4297–4309.
4. Dziuba MV, Zwiener T, Uebe R, Schüler D. Single-step transfer of biosynthetic operons endows a non-magnetotactic *Magnetospirillum* strain from wetland with magnetosome biosynthesis. Environ Microbiol. 2020;22(4):1603–18.
5. Raschdorf O, Müller FD, Pósfai M, Plitzko JM, Schüler D. The magnetosome proteins MamX, MamZ and MamH are involved in redox control of magnetite biomineralization in *Magnetospirillum gryphiswaldense*. Mol Microbiol. 2013;89:872–86.
